# Hibiscus Suspension Culture Extract Modulates Skin Metabolism and Cellular Pathways in Human Keratinocyte/Fibroblast Co-Cultures: A Proteomic Approach

**DOI:** 10.3390/biom16091243

**Published:** 2026-08-27

**Authors:** Rachid Anane, Su Melser, Elodie Renouf, Rachid Ennamany, Jean-Michel Mérillon

**Affiliations:** 1 Naolys, 86 Route d’Arcachon, 33610 Cestas-Pierroton, France; r.anane@naolys.com (R.A.); ennamany@wanadoo.fr (R.E.); 2 Elysia Bioscience, 40 Avenue Ferdinand de Lesseps, 33610 Canéjan, France; su.melser@elysia-bioscience.com; 3 Polyphenols Biotech, Equipe Molécules d’Intérêt Biologique, UMR 1366 INRAE, Faculté des Sciences Pharmaceutiques, Université de Bordeaux, 210 Chemin de Leysotte, 33140 Villenave d’Ornon, France; elodie.renouf@u-bordeaux.fr

**Keywords:** *Hibiscus syriacus*, *Hibiscus rosa sinensis*, plant cell culture, phytochemicals, polyphenols, keratinocyte/fibroblast co-culture, proteomics

## Abstract

Hibiscus plant cell cultures were developed to produce a cosmetic ingredient with anti-ageing properties. UHPLC-DAD-MS analysis of Hibiscus plant cell cultures revealed a high content of polyphenols, particularly hydroxycinnamic acid derivatives, including caffeoyl and p-coumaroyl conjugates. The biological activity of a 50/50 mixture of extracts from *Hibiscus syriacus* and *Hibiscus rosa-sinensis* cells was investigated in a human keratinocyte/fibroblast co-culture model, which better reproduces the reciprocal epithelial–mesenchymal interactions between epidermal keratinocytes and dermal fibroblasts than monocultures, using quantitative data-independent acquisition (DIA) LC-MS/MS proteomics combined with functional enrichment and protein–protein interaction analyses. A total of 7062 proteins were identified, of which 280 were differentially expressed (107 upregulated and 171 downregulated) following hibiscus treatment. The proteomic profile suggested coordinated molecular reprogramming associated with extracellular matrix remodelling, tissue repair, hydration, and attenuation of inflammatory signalling. Functional enrichment analysis revealed coordinated modulation of extracellular matrix organization, glycosaminoglycan metabolism, lysosomal function, cell communication, and inflammatory signalling. Upregulation of extracellular matrix and adhesion proteins, including lumican, collagen VIII, fibulin-5, syndecans, and glypicans, suggested coordinated extracellular matrix remodelling that may promote skin firmness and elasticity, while the downregulation of inflammatory regulators, including CARD16 and S100 family proteins, suggested attenuation of innate inflammatory responses. Overall, these findings provide mechanistic insights into the biological activity of Hibiscus cell culture extracts and support their potential as cosmetic ingredients promoting skin homeostasis and healthy skin ageing.

## 1. Introduction

The skin undergoes ageing due to the combined effects of time and environmental factors such as sunlight (UV rays). The main signs of ageing are the appearance of wrinkles, age spots, decreased elasticity, and dryness. Keratinocytes, i.e., the main connective cells of the epidermis, act as a barrier. During ageing, the epidermal barrier thins and deteriorates, leading to significant dehydration. The process of keratinocyte differentiation becomes impaired, leading to oxidative stress and inflammatory reactions. The dermis is primarily composed of fibroblasts, which synthesize major extracellular matrix components including collagen and elastin fibres. The hyaluronic acid contained in the extracellular matrix allows water to be retained. During ageing, however, fibroblast dysfunction creates an imbalance leading to degradation rather than biosynthesis. Better understanding of the biological mechanisms subsuming the ageing process could lead to new strategies to prevent this impairment and to treatments that restore a more youthful appearance to the skin, at least partially [1].

Plants biosynthesize primary metabolites such as carbohydrates, lipids and proteins, as well as a large number of secondary or specialized metabolites, mainly alkaloids, terpenes and polyphenols. All these plant compounds are of considerable importance in various fields: food and nutraceuticals, pharmaceuticals and cosmetics [2,3]. The demand for plant products is constantly growing, mainly due to the sharp increase in the world’s population. For example, food needs are expected to rise by 60% by 2050, but with an increased capacity of only 2% in agricultural land [4]. Given this socio-economic context, plant cell cultures, which have been developed since the middle of the 20th century, are an attractive alternative for the industrial production of plant compounds.

The industrial production of plant ingredients by plant cell cultures was first performed in the 1980s in Japan by the Mitsui Petrochemical Industries Company in the field of cosmetology. Mitsui produced a red dye, shikonin, from *Lithospermum erythrorhizon* cells and a skin whitening agent, arbutin, from *Catharanthus roseus* cells [5]. This breakthrough was followed by two other industrial productions of plant cell cultures in bioreactors of several tens of thousands of litres: in the 1990s, Nitto Denko Corp. (Japan) and Unhwa Biotech Corp. (South Korea) produced *Panax ginseng*, a dietary supplement containing ginsenosides; and in the 2000s, Phyton Biotech (Germany) used yew cells (*Taxus* spp.) to manufacture a precursor of paclitaxel, a major anticancer drug [6]. Recently, plant cell cultures from stoneberry, cloudberry, lingonberry, arctic bramble and birch were investigated in Finland for their nutritional and sensory properties, as well as their sustainability and safety. Altogether, the potential for using plant cell cultures as food and compound sources is growing [4,7,8].

The cosmetics industry currently represents a global market of around 450 billion euros in annual sales, of which Japan, the United States, the EU and China account for the largest share. The annual growth rate of this market is around 5%, which might lead to tension on plant product markets [9]. Plant cell cultures are of major interest for the cosmetics sector due to their capacity to biosynthesize the secondary metabolites found in vivo, at levels often higher than those of the field-grown plants, as largely shown by numerous studies carried out between the 1980s and 2000s [10]. Plant cell cultures have many advantages for the production of cosmetic ingredients in comparison to the plant *per se* [6,11]. First, they represent a production system that is independent of seasonal supply, the biotope, and climatic and putative geopolitical problems. They offer good traceability, while a plant supply chain is more difficult to control. Second, they make it possible to obtain biomass from an endangered plant, e.g., from a very slow-growing plant that is harvested in large quantities, making them an ecological, sustainable and renewable resource. Third, they have a well-controlled production system which provides constant quality irrespective of geographical areas and cultivation methods, thus avoiding the eventuality of chemical or microbial pollution. Fourth, it is easy to extract metabolites from undifferentiated cells, so the use of organic solvents is limited and physical extraction processes may be employed. Finally, unlike chemical synthesis, which is time-consuming and often costly, they make it possible to obtain complex mixtures of secondary metabolites, which themselves may often be of complex structure. Briefly, such products may be considered good candidates for meeting the requirements of good manufacturing practice.

In this study, we propose the development of plant cell cultures of hibiscus (*H. syriacus* and *H. rosa sinensis*) and the production of a cosmetic ingredient to promote healthy ageing. The phytochemical composition of *Hibiscus* has mainly been studied in plants, which contain a high content of polyphenols, especially phenolic acids and flavonoids [12,13]. Numerous studies have shown that polyphenols possess powerful antioxidant and anti-inflammatory activities, so they are considered potential anti-ageing compounds for reducing the risk of ageing-related diseases and preventing premature skin ageing [14,15]. All these activities are to be found in *Hibiscus* extracts [16,17]. To investigate the potential mechanisms underlying the biological activity of a 50/50 mixture of extracts from *Hibiscus syriacus* and *Hibiscus rosa-sinensis* cells, a proteomic approach was applied to human keratinocyte–fibroblast co-cultures. Because the biological effects of cosmetic ingredients may involve coordinated responses across multiple skin cell populations, experimental models that account for the interactions between epidermal keratinocytes and dermal fibroblasts can provide biologically relevant insights that cannot be fully captured in monocultures of either cell type alone. Keratinocytes, the predominant cell type in the epidermis, play essential roles in epidermal barrier function, differentiation, and inflammatory responses, whereas dermal fibroblasts are key regulators of extracellular matrix synthesis, organization, and remodelling, including processes involving collagen and elastin [1]. Thus, although monocultures of either cell type provide valuable cell-specific information, their co-culture enables the investigation of interconnected epidermal and dermal responses within a common experimental system. Indeed, keratinocytes and fibroblasts engage in reciprocal paracrine communication through a complex network of cytokines, chemokines, and growth factors, thereby contributing to the maintenance of skin homeostasis, epidermal differentiation, inflammatory responses, extracellular matrix remodelling, and tissue repair [18,19]. Accordingly, keratinocyte–fibroblast co-culture models constitute a relevant intermediate experimental approach between conventional monocultures and more complex three-dimensional reconstructed skin models for the preliminary evaluation and characterization of plant-derived ingredients intended for cosmetic applications [20,21,22].

## 2. Materials and Methods

### 2.1. Plant Cell Culture

Cell suspension cultures of *Hibiscus syriacus* and *Hibiscus rosa sinensis* (Malvaceae) were established in the Naolys laboratory from petioles. They were maintained under continuous fluorescent light (4500 lux) at 25 ± 1 °C in 250 mL Erlenmeyer flasks containing 50 mL of cell suspension on an orbital shaker (110 rpm). The maintenance modified Linsmaier & Skoog medium [23] contained B5 macroelements, microelements (without CoCl_2_) and vitamins and was supplemented with 58mM sucrose (100 mg/L = 555 µM), myo-inositol (0.4 mg/L = 1.19 μM), and (0.4 mg/L = 1.19 μM) Thiamine-HCl. Cells were subcultured every week by inoculating them at a 1/10 (*v*/*v*) ratio into fresh medium. For production purposes, we inoculated a 7-day-old cell suspension into the production medium at a 1/8 (*v*/*v*) ratio. This was similar to the maintenance medium, but contained 88 mM sucrose. Cells were harvested on the 12th day by vacuum filtration or centrifugation. Cells were stored at −20 °C, then lyophilized and ground [24].

### 2.2. Analysis of Phenolic Compounds

Lyophilized cells (50 mg) were extracted twice with 1 mL of solvent: first with methanol (HPLC Grade, Fisher Scientific, Loughborough, UK), followed by methanol/water (70:30; *v*/*v*). The combined supernatants (1 mL) were evaporated to dryness and reconstituted in 0.1 mL of methanol/water (70:30; *v*/*v*). The resulting solution was centrifuged and then injected into the UHPLC–MS system. All analyses were performed in triplicate.

Chromatographic analyses were performed using an Agilent 1290 Series UHPLC system (Agilent Technologies, Santa Clara, CA, USA) equipped with a UV/Vis diode array detector (DAD) and coupled to an Esquire 6000 ion trap mass spectrometer (Bruker Daltonics, Billerica, MA, USA) with an electrospray ionization (ESI) source. Chromatographic separation was achieved on a Zorbax SB-C18 column (100 × 2.1 mm i.d., 1.8 μm) with a guard column (5 × 2.1 mm i.d.) (Agilent Technologies, Santa Clara, CA, USA). The mobile phase consisted of water acidified with 0.1% formic acid (Optima LC/MS Grade, Fisher Scientific, Loughborough, UK; solvent A) and acetonitrile (Optima LC/MS Grade, Fisher Scientific, Loughborough, UK) acidified with 0.1% formic acid (solvent B), delivered at a flow rate of 0.4 mL/min using the following gradient: 1–7% B (0–2.4 min), 7–14% B (2.4–5 min), 14–18% B (5–12 min), 18–50% B (12–16 min), 50–92% B (16–18 min), 92–98% B (18–19 min), and 98% B (19–20 min). Mass spectrometric detection was performed in negative ionization mode with the following parameters: drying gas (nitrogen) flow rate, 10 L/min; nebulizer pressure, 40 psi; drying gas temperature, 365 °C; capillary voltage, 3400 V; capillary exit voltage, −115.3 V; skimmer voltage, −40 V; and trap drive, 50.7. MS data were acquired in full scan mode within the range of *m*/*z* 100 to 1450. Compound quantification was performed by UV detection at the wavelength corresponding to the maximum absorption of each analyte, and the results were expressed as equivalents of structurally related reference compounds.

To confirm molecular identities and determine molecular formulas, high-resolution mass spectrometry (HRMS) analyses were performed using a UHPLC Vanquish system (ThermoFisher Scientific, Bremen, Germany) coupled to a Q-Exactive Plus mass spectrometer (ThermoFisher Scientific) equipped with a heated electrospray ionization (HESI) source. Chromatographic separation was achieved on a Luna Omega Polar C18 column (50 × 2.1 mm, 1.6 μm; Phenomenex, Torrance, CA, USA) at 40 °C. The mobile phase consisted of water acidified with 0.1% formic acid (solvent A) and acetonitrile acidified with 0.1% formic acid (solvent B), delivered at a flow rate of 0.5 mL/min using the following gradient: 1–36% B (0–7.5 min), 36–95% B (7.5–8.5 min), and 95% B (8.5–10 min). HRMS analyses were performed in negative ionization mode with the following parameters: spray voltage, 3000 V; sheath gas flow, 45 a.u.; auxiliary gas flow, 15 a.u.; capillary temperature, 320 °C; probe heater temperature, 250 °C; and S-lens RF level, 100. Mass spectra were acquired in Full Scan mode within the range of *m*/*z* 70 to 1050 with the following parameters: resolution, 35,000; AGC target, 3 × 10^6^; maximum IT, 100 ms.

### 2.3. Human Cell Culture and Treatments

Normal human epidermal keratinocytes (NHEK.f-c) from a single donor (494Z030.2, PromoCell, Heidelberg, Germany) were cultured and maintained as a monolayer at <75% confluence in the culture medium recommended by the vendor. Keratinocytes were incubated at 37 °C under 5% CO_2_. Normal human dermal fibroblasts (NHDF-c) from a single donor (494Z030.4, PromoCell) were cultured and maintained as a monolayer at <75% confluence in the culture medium recommended by the vendor, and incubated at 37 °C under 5% CO_2_ and constant humidity. Co-culture of NHEK and NHDF cells was performed at a 1:3 seeding ratio (NHEK:NHDF). For routine passaging, cells were detached using a combination of purified trypsin and trypsin/FBS and were used up to passage 6.

NHEK:NHDF were seeded into 60 mm Petri dishes at 1 × 10^6^ total cells and incubated with the hibiscus extract at 0.5% (*v*/*v*) in the culture medium for 48 h. The extract was tested in triplicate within a single experiment, with an untreated reference control.

### 2.4. Protein Quantification and Analysis by LC-MS/MS

At the end of treatment, cells were collected, washed with PBS and solubilized in lysis buffer containing 0.5% SDS, 1% TX-100, 1% DTT and 1% Protease Inhibitor Cocktail in PBS. Suspensions were then placed at 4 °C for 1 h, followed by centrifugation at 16,000 *g* for 5 min at 4 °C. Supernatants were aliquoted and stored at −80 °C. Protein concentrations were determined using the BCA assay.

For each sample, 5 ug were migrated (SDS-PAGE) and stained with colloidal blue. Densitometric analysis of each lane was performed to adapt peptide resuspension volumes before LC-MS/MS analysis. Bands were excised from the gel and destained. Disulfide bonds were reduced, cysteine residues were alkylated, and proteins were digested overnight with trypsin. Resulting peptides were extracted from the gel and acidified prior to LC-MS/MS analysis.

Peptides were analyzed on an Orbitrap Fusion Lumos mass spectrometer (Thermo Fisher Scientific, San Jose, CA, USA) using a 146 min data-independent acquisition (DIA) method with Orbitrap detection for both MS1 and MS2 scans (OT/OT). Peptides were loaded using solvent A′ composed of H_2_O/ACN/TFA (99:1:0.1; *v*/*v*/*v*), onto a 300 µm ID × 5 mm C18 PepMap™ guard column. Chromatographic separation was performed on a 75 µm ID × 60 cm C18 Aurora Frontier™ analytical column, 1.7 µm particle size, 120 Å pore size (IonOpticks), using mobile phase A composed of H_2_O/FA (100:0.1; *v*/*v*), and mobile phase B composed of H_2_O/ACN/FA (20:80:0.1; *v*/*v*/*v*).

The raw spectra obtained were analyzed using Proteome Discoverer 3.1 with Chimerys as the search algorithm, using a Human Uniprot database UP000005640 (Reference proteome, 83,526 entries), including two missed cleavage sites by trypsin, carbamidomethylation of cysteine (+57 Da as static modification), oxidation of methionine (+16 Da as dynamic modification), acetylation of protein N-term (+42 Da as dynamic modification), methionine loss (−131 Da as dynamic modification) and methionine loss/acetylation (+89 Da as dynamic modification).

### 2.5. Bioinformatic and Statistical Analysis

Effects were assessed using a custom Python 3.13 workflow for unpaired pairwise comparisons. Raw protein abundance values were log2-transformed and normalized by centering each sample around the global median of the untreated and treatment samples. Missing values were imputed using a simple heuristic method inspired by Perseus [25], with proteins retained if they contained at least one positive abundance value in both of the two analyzed conditions before imputation. Missing values were imputed using a fixed random seed and a low-intensity normal distribution, with the imputation mean shifted 2.5 standard deviations below the observed sample mean and a distribution width of 0.3 standard deviations. Hibiscus effects are reported as log2 fold change relative to the untreated control.

Statistical testing was performed using the two-sided Welch unequal-variance *t*-test for each protein. Raw *p*-values were adjusted across proteins using the Benjamini–Hochberg false discovery rate. Proteins were considered up- or down-regulated when they met the threshold of q ≤ 0.1, *t*-test *p*-value ≤ 0.05 and the log2 fold change ≥ 0.5 or ≤−0.5. Given the number of biological replicates (*n* = 3 per condition), a post hoc sensitivity analysis was conducted to assess the robustness of the statistical design to identify the most robust protein- and pathway-level changes. Protein identifiers, standardized to the Uniprot database UP000005640, were mapped to the DAVID Knowledgebase [26] and annotation network in Cytoscape (3.10.3) using the full STRING network Homo sapiens, version 12, with a confidence score cut-off of 0.4 [27]. Unmapped identifiers were excluded from analyses.

## 3. Results

### 3.1. Characterization of Phenolic Compounds in Extracts of H. syriacus and H. rosa sinensis

**Figure 1:**
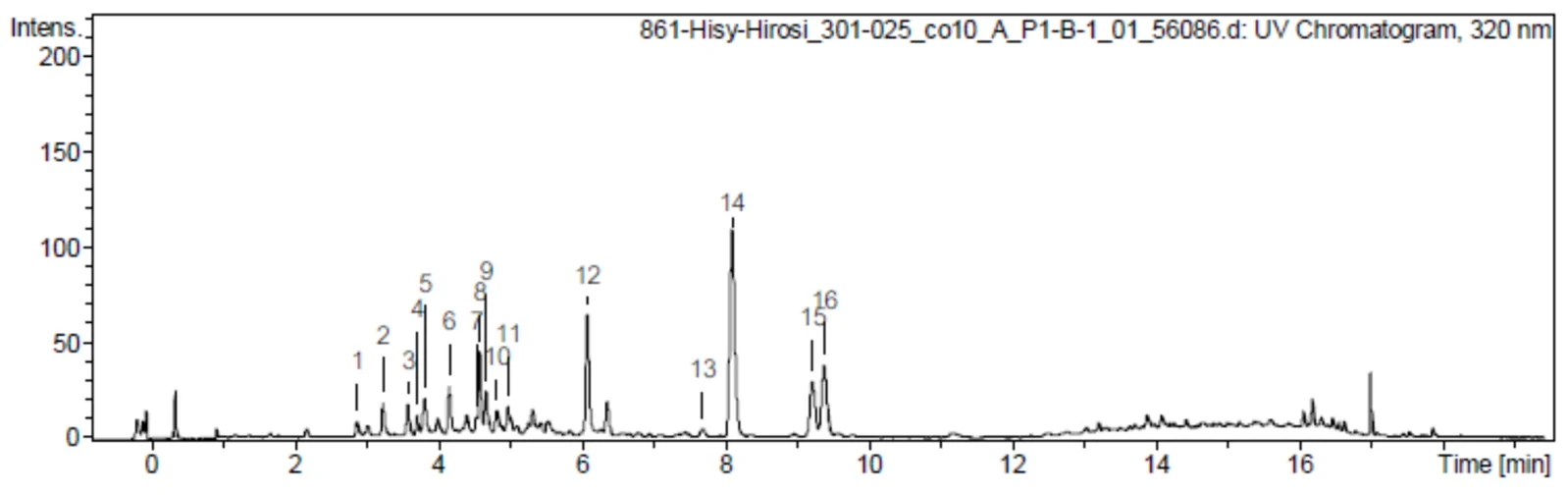
UHPLC-DAD chromatogram obtained at 320 nm from *Hibiscus* plant cell* extract (* mixture of *H. syriacus* and *H. rosa sinensis* cells 50/50, m/m).

The main polyphenols, tentatively identified in cell suspensions of *H. syriacus* and *H. rosa sinensis* using mass spectrometry, are presented in Table 1. We analyzed these two species separately, as well as a 50/50 mixture of the two species used to test biological activity. Figure 1 shows only the UHPLC-DAD chromatogram of this mixture. We observed a wide variety of phenolic acids, totalling seventeen, but no flavonoids were detected. The main polyphenols were caffeic acid derivatives (seven) and coumaric acid derivatives (eight). There was also one ferulic acid derivative and one sinapic acid derivative. Overall, the *H. syriacus* extract was richer in phenolic acids (approximately 600 μg phenolic acids/gDW) than that of *H. rosa sinensis* (approximately 250 μg/gDW). Caffeic and p-coumaric acids are well known in plants of different *Hibiscus* species [13,28,29]. These compounds, which we found in *Hibiscus* cell cultures (Table 1), are primarily caffeoyl-hexaric acids and *p*-coumaroyl-hexaric acids. The compounds we propose are as follows: three isomers of caffeoyl-glucaric acids (**1**, **2** and **5**), three isomers of *p*-coumaroyl-glucaric acids (**5**, **6** and **10**), one isomer of caffeoyl-methylglucaric acid (**4**), and two isomers of *p*-coumaroyl-methyl glucaric acids (**9** and **11**). To our knowledge, hexaric acids have never been identified in the genus *Hibiscus*, although they are known in many other plants, e.g., in *Berberis iliensis* and *B. microphylla* [30,31], in the *Asteraceae* [32,33,34], in *Syringa vulgaris* [35], in *Mercurialis perennis* [36], in the argan fruit [37], in *Solanum glaucophyllum* [38], in *Athrixia phylicoides* [39], in the *Inuleae* [40] and in the genus *Blumea* [41]. In addition, several isomers of caffeoyl-methyl glucaric acids have been identified in *Artemisia absinthium* [33].

We also found isocitric acid conjugates in *Hibiscus* cells that, to our knowledge, have never been identified in this genus (Table 1). We propose the following compounds: caffeoyl-isocitric acid (**12**), *p*-coumaroyl-isocitric acid (**14**), feruoyl-isocitric acid (**15**), and sinapoyl-isocitric acid (**16**), in agreement with the MS fragmentation patterns of hydroxycinnamoyl-isocitric acids identified by Masike et al. [42] in *Amaranthus viridis* leaves. These isocitric acid conjugates are present in much greater quantities in *H. syriacus* cells than in *H. rosa sinensis cells*, while the opposite is true for the other caffeoyl and coumaroyl derivatives. Therefore, we created a 50/50 mixture of cells from the two species to obtain a balanced extract containing all the phenolic acids necessary for biological activities.

Calluses and cell suspensions of *Hibiscus cannabinus* have been obtained for the production of exopolysaccharides [43,44], as well as cultures of *Hibiscus sabdariffa* calluses for the production of anthocyanins [45]. Di Martino et al. [46] established cell suspensions of *Hibiscus syriacus* accumulating coumarins and flavonoids, which were neither characterized nor quantified. However, an ethanolic extract of these cells interestingly demonstrated stimulation of skin wound healing using human fibroblasts, keratinocytes, and skin explants. Calluses of the same species were obtained by Xu et al. [47], but the production of specialized metabolites was not studied. An ethanolic extract of these cells possessed prominent anti-inflammatory activity.

### 3.2. Protein Quantification and Analysis by LC-MS/MS After Treatment of Keratinocyte/Fibroblast Human Co-Culture by H. syriacus and H. rosa sinensis Extract

**Figure 2:**
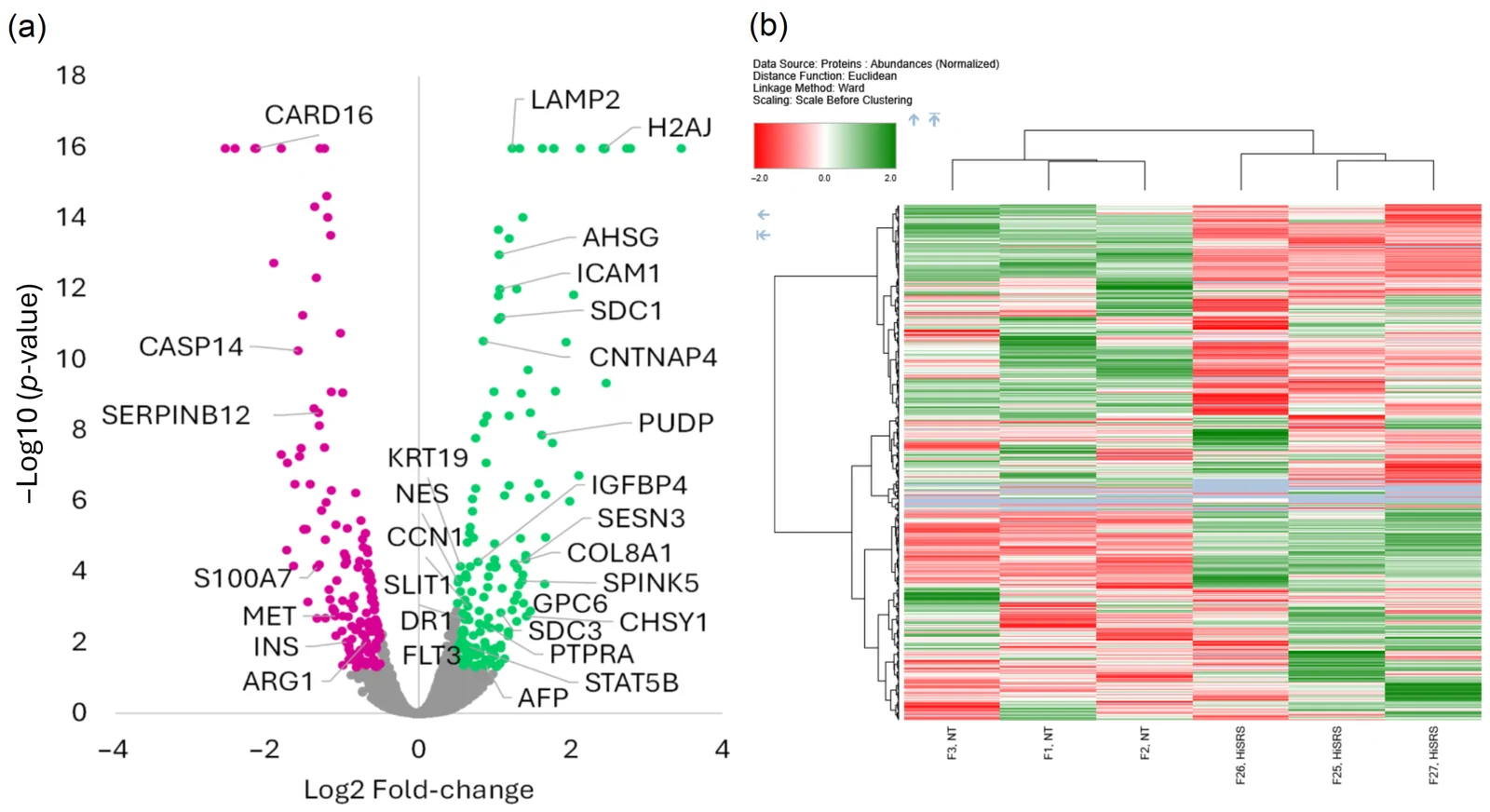
(**a**) Volcano plot displaying the differentially expressed proteins (DEPs) in NHEK/NHDF co-cultures treated with hibiscus (HiSRS) compared to the untreated control group. The *X*-axis represents the expression fold change (Log_2_ Ratio HiSRS/NT), and the *Y*-axis represents the *p*-value (−log_10_ *p*-value). Magenta dots indicate upregulated DEPs, while cyan dots represent downregulated DEPs. Grey dots denote proteins whose expression did not differ between the groups. The threshold for statistical significance was set at a *p*-value < 0.05. (**b**) Heatmap showing the DEPs in NHEK/NHDF co-cultures treated with hibiscus (HiSRS) compared to the control group (NT). Protein abundance data were normalized and scaled after clustering. Euclidean distance and complete linkage were employed as the clustering method. Each row represents a protein, and each column represents an experimental sample. Colours indicate relative expression levels: green for high expression, white for medium expression, light blue for absence, and red for low expression. The dendrograms at the top and left illustrate the similar relationships among samples and proteins, respectively.

LC–MS/MS identified a total of 7062 proteins. The heatmap (Figure 2b) highlights the distinct expression patterns between treated and control groups after hierarchical clustering of the proteins identified.

Functional enrichment analysis was performed with DAVID (2021 update) to assign biological meaning to the proteomics data. After identifier mapping, 6813 of the 7062 MS-identified proteins were retained for annotation. Enrichment results were summarized across curated annotation sources (Gene Ontology and pathway databases), and statistically enriched terms (multiple-testing corrected) were used to define dominant relevant biological processes and pathways associated with the dataset.

For network-based analysis in Cytoscape, protein identifiers were mapped to version-12.string-db.org identifiers, with a cut-off of 0.4. In total, 6621 out of the initial 7062 proteins (93%) mapped successfully and were retained for protein–protein interaction analysis. Overall, the IDs that were not represented in the annotation knowledge base and protein–protein interaction networks under the selected database and confidence settings are consistent with known limitations when translating MS-derived protein outputs into functional protein information.

Based on statistics, a total of 280 differentially expressed proteins (DEPs) were identified, comprising 107 upregulated and 171 downregulated proteins. These findings are illustrated in the volcano plot (Figure 2a), showing protein expression changes (Log2 Ratio) against statistical significance (−log10 *p*-value), and the dendrogram (Figure 2b) showing the distinct expression patterns.

**Figure 3:**
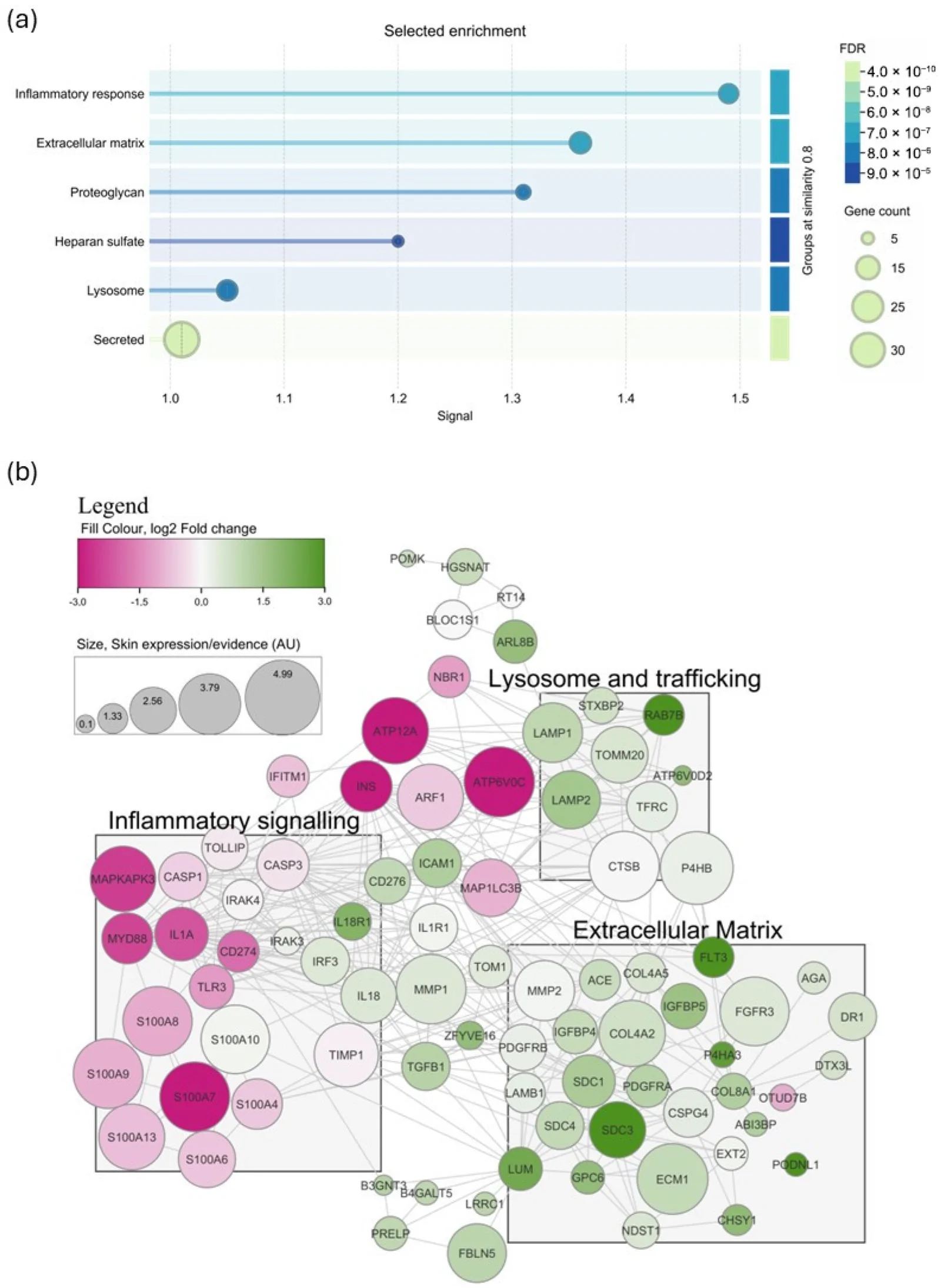
(**a**) GO annotation of the cellular component terms of DEPs versus their fold enrichment, performed using the DAVID and STRING databases with homo sapiens proteome as a background. (**b**) Visualization of the protein–protein interaction (PPI) network of selected proteins using STRING. The clusters reflect the identified enriched biological functions and pathways. PPI enrichment *p*-value < 1.0 × 10^−16^.

DAVID GO enrichment and protein–protein interaction networks of total annotated proteins analyzed from NHEK:NHDF co-cultures treated for 48 h with hibiscus helped identify 103 out of 107 proteins increased in abundance versus untreated controls (Log2 fold-change range 1.00–3.86). Among the up-regulated proteins, 29 out of 103 increased ≥4-fold (log2 fold-change ≥ 2). The strongest individual responses included broad remodelling of extracellular and endo-lysosomal components (Figure 3a). Also identified were 168 annotated proteins out of 171 proteins decreased in abundance versus untreated controls (Log2 Fold-change range −0.83 to −5.02). Among the down-regulated proteins, 60 out of 168 proteins decreased ≥4-fold. The strongest individual responses included cornified envelope and keratin components.

Extracellular matrix and collagen-containing matrix signatures were prominent among the up-regulated proteins. Multiple structural or matrix-associated factors were up-regulated, including lumican (LUM, log2 fold-change 2.4), collagen VIII (COL8A1; log2 fold-change 1.2), fibulin-5 (FBLN5; log2 fold-change 1.0), extracellular matrix protein 1 (ECM1; log2 fold-change 0.9), and podocan-like 1 (PODNL1, log2 fold-change 3.2). The extracellular matrix (ECM) is widely recognized as a complex structure of macromolecules, including molecules derived from small leucine-rich proteoglycans (SLRPs), which are crucial for tissue development, homeostasis, and pathological processes [48,49]. Lumican is a small leucine-rich proteoglycan with well-established roles in collagen fibrillogenesis and connective tissue integrity, including skin [50]. In lumican-deficient mice, abnormal collagen fibril assembly is associated with skin fragility, directly supporting its relevance to dermal matrix architecture [51]. B4GALT5 (log2 fold-change 1.0), which catalyzes the synthesis of lactosylceramide (LacCer), can affect permeability differentiation in the basal keratinocyte layers [52,53]. ECM1 has been described as an organizer of skin extracellular matrix and basement membrane-associated architecture [54], with links to collagen assembly and growth factor-binding interactions. Together, these changes are consistent with the reinforcement and/or re-organization of collagen-containing extracellular matrix in the co-culture.

Two cell-surface heparan sulphate proteoglycans (HSPGs) were also increased, syndecan-3 (SDC3, log2 fold-change 3.6) and syndecan-1 (SDC1, log2 fold-change 1.4), along with glypican-6 (GPC6, log2 fold-change 1.8). Syndecan-1 is a well-characterized HSPG capable of binding a wide range of extracellular matrix ligands, cytokines, and growth factors via heparan sulphate chains, and it has established roles in repair processes such as cell adhesion, migration, and ECM assembly. Glypican-6 has been linked to modulation of growth factor signalling at the cell surface [55,56]. In parallel, chondroitin sulphate synthase 1 (CHSY1, log2 fold-change 1.8) supports a shift in glycosaminoglycan (GAG) synthesis, consistent with increased proteoglycan/GAG production and remodelling [57,58].

Increased abundance of lysosome-associated proteins indicated coordinated engagement of the endo-lysosomal system (Figure 3b). LAMP1 (log2 fold-change 1.0) and LAMP2 (log2 fold-change 1.5) are canonical lysosomal membrane glycoproteins and, together, were up-regulated. ARL8B, ATP6V0D2, and ZFYVE16/endofin support activation of the endolysosomal system, particularly lysosomal trafficking, positioning, fusion and V-ATPase-linked lysosomal function [59,60,61]. Furthermore, heparan-alpha-glucosaminide N-acetyltransferase (HGSNAT, log2 fold-change 0.8), which catalyzes a key lysosomal step required for heparan sulphate degradation [58], suggests enhanced turnover/processing of heparan sulphate-containing substrates.

Finally, the increases in insulin-like growth factor-binding protein 5 (IGFBP5, log2 fold-change 1.6), IGFBP4 (log2 fold-change 0.9) and intercellular adhesion molecule 1 (ICAM1, log2 fold-change 1.1) further support a plausible effect of hibiscus on matrix remodelling and repair programmes. IGFBP5 has been shown in vivo to promote collagen deposition and dermal thickening after overexpression in a murine skin model [62], and it may be involved in epithelial and fibroblast changes consistent with fibrotic responses [63]. Other up-regulated receptor and adhesion signalling proteins such as PDGFRA and FLT3 are also consistent with broad modulation of intercellular communication and biomechanical properties in the co-culture microenvironment. In particular, PDGF/PDGFRA signalling is linked to fibroblast activation, migration and extracellular matrix remodelling [64], while FLT3/FLT3L signalling may reflect cell communication [65].

Several immune/innate defence signalling proteins were down-regulated, including S100 family members (S100A8 and S100A7/psoriasin), which are commonly linked to epithelial innate defence and inflammatory skin programmes [66]. Caspase recruitment domain-containing protein 16 (CARD16, log2 fold-change −5.0) was also down-regulated by hibiscus treatment. CARD16 acts as a regulator of procaspase- 1/CASP1 activation and is involved in the regulation of the proteolytic maturation of pro-interleukin-1 beta (IL1B) and its release during inflammation [67]. The adjacent protein-protein network also detected IL1A, TLR3 and MYD88 as being potentially decreased. IL1A is a cytokine family member with well-described roles in amplifying inflammation via keratinocyte-driven innate immune cascades [68], whereas MYD88 is a central adaptor for IL-1 receptor and most TLRs [69]. IL1A, TLR3 and MYD88 converge on inflammatory transcription programmes [70]. Overall, the down-regulated proteins suggest an effect of hibiscus on the regulation of immune-related responses with anti-inflammatory potential.

## 4. Discussion

Plant cell suspension cultures constitute an attractive biotechnology platform because they not only provide a sustainable and reproducible source of plant-derived biomolecules but also generate specialized metabolite profiles that frequently differ from those found in differentiated plant tissues. In the present study, suspension cultures of *Hibiscus syriacus* and *Hibiscus rosa-sinensis* were developed to produce a standardized extract rich in phenolic compounds. Phytochemical characterization revealed that hydroxycinnamic acid derivatives, particularly caffeoyl- and *p*-coumaroyl-conjugates, represent the predominant class of metabolites accumulated by these cultures, together with lower amounts of feruloyl- and sinapoyl-derived compounds.

Interestingly, several caffeoyl- and *p*-coumaroyl-glucaric acid derivatives, as well as hydroxycinnamoyl-isocitric acid conjugates, were identified. To our knowledge, these metabolites have not previously been reported in the genus *Hibiscus*. Their predominance suggests that cellular dedifferentiation and in vitro cultivation redirect phenylpropanoid metabolism toward specific hydroxycinnamate conjugates that are not commonly accumulated in differentiated plant tissues. Hydroxycinnamic acids, particularly caffeic, *p*-coumaric, ferulic, and sinapic acid derivatives, are widely recognized for their antioxidant, anti-inflammatory, and photoprotective properties, making them particularly relevant for skin protection and healthy skin ageing [14,15].

The quantitative proteomic analysis performed on human keratinocyte/fibroblast co-cultures provided valuable insight into the molecular mechanisms underlying the biological activity of the hibiscus extract. More than 7000 proteins were identified, providing extensive proteome coverage and enabling a systems-level evaluation of the cellular response. Rather than affecting a limited number of isolated proteins, the extract induced coordinated modulation of biological pathways involved in extracellular matrix organization, glycosaminoglycan metabolism, cellular communication, lysosomal function, and inflammatory signalling.

One of the most prominent findings is the enrichment of proteins associated with extracellular matrix organization and collagen-containing structures. Increased abundance of lumican (LUM), fibulin-5 (FBLN5), collagen VIII (COL8A1), extracellular matrix protein 1 (ECM1), and podocan-like protein 1 (PODNL1) is consistent with coordinated extracellular matrix remodelling involving structural, matricellular, and collagen-associated proteins. Lumican is a key regulator of collagen fibrillogenesis and contributes to dermal integrity and mechanical strength, whereas fibulin-5 plays a central role in elastic fibre assembly and maintenance of skin elasticity. Collectively, these proteomic changes suggest that hibiscus extract promotes an extracellular environment favourable to matrix maintenance, tissue integrity, and structural resilience.

The upregulation of syndecans, glypicans, and chondroitin sulphate synthase further supports this interpretation. Beyond their structural role in glycosaminoglycan biosynthesis, syndecans and glypicans act as major organizers of the extracellular signalling environment by regulating the spatial presentation of growth factors to their receptors. These mechanisms are known to modulate key developmental pathways, including WNT, NOTCH, FGF, and TGF-β signalling, which collectively govern epidermal renewal, fibroblast activity, extracellular matrix remodelling, and tissue regeneration. Consequently, the coordinated increase in syndecans and glypicans observed following hibiscus treatment suggests reconstruction of an extracellular signalling niche that promotes controlled tissue regeneration rather than uncontrolled proliferative activation. Moreover, because glycosaminoglycans are essential for water retention and maintenance of skin turgor, modulation of these pathways may also contribute to improved hydration-related functions.

Another important observation concerns the activation of lysosomal and endo-lysosomal pathways. Increased expression of LAMP1, LAMP2, HGSNAT, ARL8B, ATP6V0D2, and related proteins suggests enhanced intracellular turnover and recycling mechanisms. Controlled lysosomal activity is increasingly recognized as a hallmark of cellular homeostasis and healthy ageing. Efficient degradation and recycling of damaged macromolecules contribute to tissue renewal, maintenance of proteostasis, and preservation of cellular functionality. Therefore, the observed modulation may reflect an adaptive remodelling process that supports skin regeneration.

Hydroxycinnamic acids are well recognized for their antioxidant properties and their ability to reduce intracellular reactive oxygen species (ROS). Because oxidative stress represents one of the major drivers of inflammaging, a chronic low-grade inflammatory state that progressively develops during ageing and contributes to tissue dysfunction, reduced ROS production is expected to attenuate redox-sensitive inflammatory pathways, including IL-1- and HIF-1-dependent signalling. Although intracellular ROS levels were not directly measured in the present study, the coordinated modulation of inflammatory proteins together with the well-established antioxidant activity of hydroxycinnamic acids is consistent with attenuation of oxidative inflammatory stress. As ROS act upstream of IL-1- and HIF-1-mediated inflammatory responses, reduction in oxidative stress may contribute to limiting inflammasome activation and subsequent amplification of inflammatory signalling.

The proteomic profile also revealed coordinated modulation of proteins involved in innate immunity and inflammatory signalling. Several inflammation-associated proteins, including members of the S100 family and CARD16, were downregulated. S100A7 and S100A8 are well-established markers of inflammatory skin disorders and epithelial stress, and their reduced abundance is consistent with attenuation of pro-inflammatory signalling. Likewise, decreased CARD16 expression, a regulator of inflammasome activation and interleukin-1 maturation, suggests reduced activation of inflammasome-associated inflammatory pathways. These observations agree with previous reports describing the anti-inflammatory properties of hibiscus-derived extracts and hydroxycinnamic acid-rich phytochemical preparations.

An additional aspect emerging from the proteomic profile is its relationship with inflammaging. In the skin, inflammaging is characterized by persistent activation of innate immune pathways, sustained production of pro-inflammatory cytokines, extracellular matrix degradation, and impaired regenerative capacity. The coordinated downregulation of inflammatory proteins, including S100A7, S100A8, and CARD16, together with activation of extracellular matrix remodelling pathways, is consistent with the attenuation of several molecular hallmarks of skin inflammaging. This dual activity, combining structural reinforcement of the extracellular matrix with reduction in inflammatory signalling, is particularly relevant to longevity-oriented skin care strategies, in which preservation of tissue homeostasis is increasingly recognized as a key determinant of healthy skin ageing.

Within these observations, CASP-1 down-regulation could be associated with decreased inflammasome-associated cell-death pathways, including canonical pyroptosis, particularly in the context of UV-induced skin stress and photo-ageing. Pyroptosis has recently emerged as an important contributor to skin inflammaging because inflammasome activation amplifies IL-1β production, promotes chronic sterile inflammation, and accelerates extracellular matrix degradation. Consequently, attenuation of this inflammatory cascade may represent one of the principal mechanisms through which hibiscus-derived metabolites contribute to maintaining tissue homeostasis.

Taken together, the proteomic data indicate that hibiscus cell culture extract does not simply target a single biological process but instead induces coordinated modulation of extracellular matrix remodelling, cellular renewal, glycosaminoglycan metabolism, lysosomal activity, and inflammatory signalling. Such a multifactorial mode of action is particularly relevant to skin ageing, which results from the interplay between structural degradation, impaired repair mechanisms, oxidative stress, and chronic low-grade inflammation.

Collectively, our data support a mechanistic model in which hydroxycinnamate-rich metabolites produced by hibiscus suspension cultures orchestrate coordinated attenuation of oxidative and inflammatory stress, thereby limiting inflammasome activation and possibly pyroptosis, while simultaneously promoting extracellular matrix reconstruction through syndecan- and glypican-mediated organization of extracellular signalling. This coordinated biological reprogramming may ultimately promote restoration of skin homeostasis.

One major strength of the present study lies in the application of quantitative data-independent acquisition (DIA) proteomics, which provides a systems-level view of the biological activity of complex botanical extracts. Rather than focusing on a limited number of predefined biomarkers, this approach captures coordinated molecular responses across interconnected biological pathways. The present findings therefore demonstrate that the biological activity of plant-derived ingredients should be interpreted in terms of network-wide molecular reprogramming rather than isolated molecular targets.

Finally, this work highlights the value of combining plant cell culture biotechnology with quantitative proteomics to elucidate the mechanisms of action of plant-derived biomolecules. Future studies should determine the contribution of individual hydroxycinnamate conjugates to the coordinated biological response observed in the present work and validate the proposed mechanisms using complementary functional approaches, including intracellular ROS quantification, inflammasome activation, extracellular matrix deposition, and ex vivo human skin models. Such investigations will help establish causal relationships between the unique phytochemical profile generated by *Hibiscus* suspension cultures and the molecular pathways governing skin homeostasis.

### Study Limitations and Interpretive Considerations

The conclusions drawn here rest on coordinated, pathway-level changes rather than isolated proteins, a deliberate emphasis on robust, systems-level signals. The convergence of multiple independent nodes reinforces confidence in the proposed mechanisms. For inflammation, the concerted decrease in CARD16, S100A7/S100A8 and network-associated IL1A, TLR3 and MYD88, which converge on shared inflammatory programmes [68,69,70], provides consistent evidence of an anti-inflammatory, inflammasome-limiting effect, fully in line with the antioxidant, ROS-lowering activity of the extract’s predominant hydroxycinnamates [14,15] and with prior reports for Hibiscus syriacus [46,47]. Likewise, the simultaneous up-regulation of LAMP1, LAMP2, ARL8B, ATP6V0D2, ZFYVE16 and HGSNAT across membrane, acidification, trafficking and degradative steps points to genuine engagement of a homeostatic endo-lysosomal/autophagic programme supporting proteostasis and renewal [71]. As these steady-state proteomic signatures are inherently associative, they establish a strong mechanistic framework that further functional work, including dose–response and time-course proteomics, inflammasome-challenge and autophagic-flux assays, and ex vivo skin models, could further substantiate and extend [72,73,74].

## 5. Conclusions

This study demonstrates that suspension cultures of *Hibiscus syriacus* and *Hibiscus rosa-sinensis* constitute an effective and sustainable source of bioactive cosmetic ingredients. The extract obtained from these cultures has a high content of hydroxycinnamic acid derivatives, including several caffeoyl-, coumaroyl-, feruloyl-, and sinapoyl-conjugates that may contribute to its biological activity.

Proteomic analysis of human keratinocyte/fibroblast co-cultures revealed extensive modulation of cellular pathways associated with extracellular matrix organization, glycosaminoglycan metabolism, lysosomal function, tissue remodelling, and inflammation regulation. The overall response suggests a coordinated biological state promoting matrix maintenance, cellular renewal, hydration-related processes, and control of inflammatory signalling.

These findings provide mechanistic evidence supporting the cosmetic potential of hibiscus plant cell culture extracts as skin longevity active ingredients. Beyond their demonstrated biological effects, plant cell cultures offer significant advantages in terms of sustainability, traceability, reproducibility, and environmental conservation. Consequently, hibiscus cell culture extracts are promising candidates for the development of next-generation cosmetic products targeting skin ageing and the maintenance of skin homeostasis.

Furthermore, the production of these active ingredients through plant cell culture technology offers a sustainable, traceable and environmentally responsible alternative to conventional botanical sourcing. This approach is fully aligned with current expectations for eco-designed cosmetic ingredients and supports the development of innovative natural products combining efficacy, reproducibility and responsible production.

## Figures and Tables

**Table 1:** Identification and quantification of main compounds present in extracts from suspension cell cultures of *Hibiscus syriacus (Hisy)* and *Hibiscus rosa sinensis (Hirosi)* using UHPLC–DAD–MS.

No	Rt (min)	Molecular Formula	[M − H]^−^ Measured	(*m*/*z*) Predicted	Δm (ppm)	ESI-MS/MS (*m*/*z*)	Proposed Compound	Contents (µg/g DW ± SD)		
								Hisy-Hirosi *	Hirosi	Hisy
1	2.9	C_15_H_16_O_11_	371.0614	371.0609	1.38	209.0294, 191.0182	Caffeoyl-glucaric acid **^(a)^	5.2 ± 0.5	n.d.	6.6 ± 0.5
2	3.3	C_15_H_16_O_11_	371.0613	371.0609	1.22	209.0292, 191.0184	Caffeoyl-glucaric acid **^(a)^	11.5 ± 1.6	n.d.	18.6 ± 4.1
3	3.6	C_14_H_16_O_9_	327.0717	327.0711	1.92	165.0390	Caffeoyl derivative **^(a)^	11.5 ± 0.2	19.2 ± 1.1	7.1 ± 0.7
4	3.8	C_16_H_18_O_11_	385.0771	385.0765	1.35	223.0450, 205.0338, 125.0231	Caffeoyl-methyl glucaric acid **^(a)^	6.5 ± 0.1	12.6 ± 0.7	2.4 ± 0.2
5	3.8	C_11_H_12_O_2_N_2_	203.0816	203.0815	0.64	159.0913, 142.0648, 116.0490	Tryptophan ***^(b)^	241.1 ± 9.4	171.5 ± 9.0	279.6 ± 23.2
		C_15_H_16_O_11_	371.0614	371.0609	1.38	209.0292, 191.0185	Caffeoyl-glucaric acid **^(a)^		n.d.	
		C_15_H_16_O_10_	355.0666	355.0660	1.74	209.0289, 191.0184	*p*-Coumaroyl-glucaric acid **^(c)^		n.d.	
6	4.2	C_15_H_16_O_10_	355.0666	355.0660	1.65	209.0293, 191.0185	*p*-Coumaroyl-glucaric acid **^(c)^	14.8 ± 1.3	n.d.	21.5 ± 2.1
7	4.6	C_14_H_16_O_9_	327.0717	327.0711	1.82	165.0391	Caffeoyl derivative **^(a)^	3.4 ± 0.3	4.0 ± 0.1	3.2 ± 0.3
8	4.6	C_14_H_16_O_8_	311.0768	311.0761	1.95	163.0386, 119.0486	*p*-coumaric acid derivative **^(c)^	22.7 ± 0.4	44.8 ± 1.5	10.4 ± 0.5
9	4.7	C_16_H_18_O_10_	369.0819	369.0816	0.86	223.0446, 205.0341, 125.0229	*p*-Coumaroyl-methyl glucaric acid **^(c)^	13.0 ± 0.3	26.5 ± 1.2	3.5 ± 0.2
10	4.8	C_15_H_16_O_10_	355.0666	355.0660	1.65	209.0296, 191.0186	*p*-Coumaroyl-glucaric acid **^(c)^	6.7 ± 0.4	n.d.	8.0 ± 0.5
11	5.0	C_16_H_18_O_10_	369.0822	369.0816	1.44	223.0448, 205.0335	*p*-Coumaroyl-methyl glucaric acid **^(c)^	6,1 ± 0,4	6.8 ± 0.5	6.3 ± 0.5
12	6.1	C_15_H_14_O_10_	353.0508	353.0503	1.30	191.0190, 173.0078, 154.9971, 111.0071	Caffeoyl-isocitric acid **^(a)^	51.1 ± 7.0	8.8 ± 0.6	74.0 ± 11.0
13	7.7	C_9_H_8_O_3_	163.0388	163.0390	−1.21	119.0486	*p*-Coumaric acid ***^(c)^	4.4 ± 0.3	n.d.	6.8 ± 0.1
14	8.1	C_15_H_14_O_9_	337.0559	337.0554	1.48	173.0081, 154.9977, 111.0072	*p*-Coumaroyl-isocitric acid **^(c)^	105.8 ± 3.1	31.2 ± 2.2	162.9 ± 2.5
15	9.2	C_16_H_16_O_10_	367.0663	367.0663	1.01	173.0078, 154.9973, 111.0070	Feruoyl-isocitric acid **^(d)^	40.9 ± 0.3	n.d.	65.4 ± 0.8
16	9.4	C_17_H_18_O_11_	397.0768	397.0765	0.77	173.0077, 154.9971, 111.0072	Sinapoyl-isocitric acid **^(e)^	55.4 ± 0.8	n.d.	93.1 ± 3.7

n.d.: not detected. * mixture of Hisy and Hirosi cells 50/50 (m/m). ** Identification of compounds based on exact mass and fragment ions. *** Chemical structure confirmed by a commercial standard. ^(a)^ Quantified in caffeic acid equivalent at 320 nm. ^(b)^ Quantified in tryptophan equivalent at 280 nm. ^(c)^ Quantified in *p*-coumaric acid equivalent at 310 nm. ^(d)^ Quantified in ferulic acid equivalent at 320 nm. ^(e)^ Quantified in sinapic acid equivalent at 320 nm. Caffeic acid, L-tryptophan, *p*-coumaric acid, ferulic acid, sinapic acid were obtained from Sigma Aldrich (St Louis, MO, USA).

## Data Availability

The data presented in this study are available on request from the corresponding author.
